# Using period analysis to timely assess and predict 5‐year relative survival for colorectal cancer patients in Taizhou, eastern China

**DOI:** 10.1002/cam4.5220

**Published:** 2022-09-05

**Authors:** Min Zhang, Yongran Cheng, Bicheng Chen, Runhua Li, Xiyi Jiang, Liangyou Wang, Tianhui Chen, Qi Liao, Jinfei Chen

**Affiliations:** ^1^ School of Public Health Hangzhou Medical College Hangzhou China; ^2^ Department of Oncology First Affiliated Hospital of Wenzhou Medical University Wenzhou China; ^3^ Department of Cancer Prevention/Zhejiang Cancer Institute, Cancer Hospital of the University of Chinese Academy of Sciences (Zhejiang Cancer Hospital); Institute of Basic Medicine and Cancer (IBMC), Chinese Academy of Sciences Hangzhou China; ^4^ Department of Non‐communicable Chronic Disease Control and Prevention Taizhou Center for Disease Control and Prevention Taizhou China; ^5^ Department of Preventative Medicine, School of Medicine Ningbo University Ningbo China

**Keywords:** cancer registry, colorectal cancer, period analysis, screening, survival

## Abstract

**Introduction:**

While timely assessment of long‐term survival for patients with colorectal cancer (CRC) is essential for evaluation on early detection and screening programs of colorectal cancer, those data are extremely scarce in China. We aimed to timely and accurately assess long‐term survival for CRC patients in eastern China.

**Methods:**

Patients diagnosed with CRC during 2004–2018 and followed up until December 31, 2018 from four cancer registries with high‐quality data from Taizhou, eastern China were included. Period analysis was used to calculate 5‐year relative survival (RS) for overall and the stratification by sex, age at diagnosis and region. The projected 5‐year RS of CRC patients during 2019–2023 was also assessed using a model‐based period analysis.

**Results:**

Overall 5‐year RS for patients with CRC during 2014–2018 reached 78.8%, being 74.9% for men and 86.1% for women. 5‐year RS declined along with aging, decreasing from 84.1% for age < 45 years to 48.9% for age > 74 years, while 5‐year RS for urban area was higher compared to rural area (83.9% vs. 75.8%). Projected overall 5‐year RS of CRC patients could reach 85.9% during the upcoming period 2019–2023.

**Conclusions:**

We provided, for first time in China using period analysis, most up‐to‐date 5‐year RS for patients with CRC from Taizhou, eastern China and also found 5‐year RS for CRC patients have improved greatly during 2004–2018.

## Graphical abstract

Period analysis is closest to the real 5‐year survival. No studies predicted the 5‐year relative survival trend of colorectal cancer in China using period analysis. An upward trend of 5‐year relative survival for colorectal cancer patients in gender, age at diagnosis, and region in Taizhou City, eastern China.

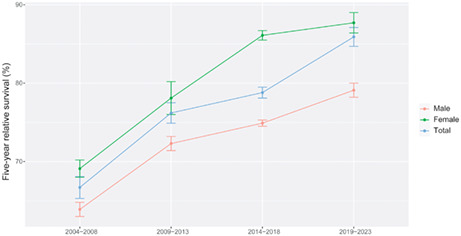

## 1 INTRODUCTION

Colorectal cancer (CRC) is a common malignant tumor in China. Early symptoms of CRC are not obvious. As the neoplasm grows up, symptoms appear, such as bloody stool, diarrhea, constipation, abdominal pain, or organ metastasis, etc. Finally, the diagnosis for CRC can be confirmed by colonoscopy and pathological tests. Adenocarcinoma is the most common pathological type for CRC, accounting for about 95%. Pathological staging standards can not only guide clinicians to determine treatment options, but also judge the prognosis of CRC patients.^1^ The global incidence and mortality of CRC are on the rise worldwide. According to the global cancer data in 2018, CRC is the third common malignant tumor in the world. The upward trend of CRC relates to diet and lifestyle habits. Obesity, sedentary lifestyle, high fat, high protein, fried food, pickled diet, as well as alcohol and tobacco are considered to cause CRC. CRC has obvious geographical distribution. In recent years, the incidence of CRC in Africa, Asia, Latin America and Europe has been on the rise.^2^

In 2018, the global new cases of CRC were 1,849,518, accounting for 10.2% of total tumor cases, and the age‐standardized incidence rate was 19.7 per 100,000, ranking third. The incidence of CRC in China was 521,490, accounting for 12.2% in Chinese cancer cases, and the age‐standardized incidence rate was 23.7 per 100,000, ranking second behind lung cancer in China.^3^ In 2018, the number of global CRC deaths was 880,792, accounting for 9.2% of cancer deaths, and the age‐standardized mortality was 8.9 per 100,000. The number of CRC deaths in China was 247,563, accounting for 8.6% in Chinese cancer deaths, and the age‐standardized death rate was 10.9 per 100,000, ranking fifth among Chinese tumor mortality.^3^ The incidence of female CRC in the UK was 1.4 times higher than that in China, but the mortality rate in the UK was 0.9 times lower than that in China in 2018. Therefore, early screening and effective treatment in developed countries are the reasons for the reduced mortality of CRC. Over the past few decades, declining CRC incidence and mortality have showed in both men and women with the widespread use of colonoscopy. Importantly, declines in overall CRC incidence mask an increase in incidence under 65‐year adults. In 2018, CRC mortality was the second leading cause for men aged 20 to 39 years and it also the leading cause for men under 50 years.^4^

Zhejiang Province belongs to the eastern China, and the incidence and mortality of CRC are higher than the other regions in China. In 2016, the incidence was 41.75/100,000 and the mortality was 17.02/100,000. In addition, the incidence and mortality in men was higher than that in women.^5^ Taizhou is a major city in Zhejiang Province, and it is also an area with a high incidence of CRC.

In the past 20 years, the global survival trend of CRC has been increasing steadily, especially in Asia, Europe and Oceania, and the survival varies greatly across the world. Between 1995–1999 and 2000–2014, the CRC survival increased by more than 10% in China, Israel and Korea, and by 5%–10% in Japan, Taiwan, Australia, Italy, Ireland, Lithuania, Norway, etc. For patients diagnosed during 2010–2014, the five‐year survival in most countries was between 50%–69%, and in Israel, Jordan, South Korea and Australia survival it was higher than 70%, and the survival was below 50% in Ecuador, Thailand, Russia and India.^6^

The 5‐year survival for CRC in China is increasing. According to the data from 17 Chinese population‐based cancer registries, the age‐standardized 5‐year relative survival (RS) in 2003–2005, 2006–2008, 2009–2011, and 2012–2015 were 47.2%, 52.7%, 52.7%, and 56.9%, showing a slowly upward trend. The survival for Chinese women was higher than Chinese men. The survival for rural patients was lower than urban patients, and the survival gap between rural and urban patients was narrowing from 2012 to 2015.^7^ The survival in Zhejiang Province is higher than the average in China. In 2005–2010, the 5‐year RS of CRC patients in Zhejiang Province was 58.73%, while the survival for colon cancer patients (61.47%) was higher than rectal cancer patients (56.45%), and the survival in urban areas (64.09%) was higher than that in rural areas (55.16%).^8^

At present, the methods for evaluating the long‐term survival worldwide are mainly cohort method, complete analysis and period analysis, which reflects the medical level and tumor prognosis in a region. Recent studies have shown the 5‐year RS calculated by period analysis is closest to the real 5‐year survival.^9^ Analyzing the precise survival is a major scientific and technological problem that urgently needs to be solved in China. Therefore, period analysis was used in this paper to assess the overall and stratified data on the 5‐year RS for CRC in Taizhou City, Zhejiang Province, eastern China.

## 2 MATERIALS AND METHODS

### 2.1 Data source

Taizhou city with 6.6 million inhabitants is covering roughly 10% population of Zhejiang province, which is located at the eastern coast of Zhejiang province and approximately 300 km away from southern Shanghai, China. Case data were retrieved from 9 cancer registries in Taizhou from 2004 to 2018. It is acceptable to evaluate the quality of the data with the proportion of death certificate (DCO) cases less than 13% of all cancer cases. Therefore, data from the four cancer registries (Luqiao, Yuhuan, Xianju, and Wenling) were included for the further analysis. As of December 31, 2018, the follow‐up investigation of the life condition has been completed.

According to the tenth edition of the International Classification of Diseases (ICD‐10), the cases coded C18‐C20 were identified as colorectal cancer patients. Therefore, a total of 10,966 colorectal cancer patients were initially confirmed. In addition, 1120 without a clear time of diagnosis, 180 without a histological diagnosis, 428 cases of logic errors and 752 patients who were lost to follow‐up last time were excluded. In the end, a total of 8486 patients were left for further analysis.

The data quality was high because the data were obtained from government agencies, adopted the international classification standard for colorectal cancer, and used strict inclusion and exclusion criteria. The percentage of histology‐based diagnoses was 98.4% and 6.9% of the cases lost follow‐up. All these ensure the authenticity and validity of the data.

### 2.2 Statistical analysis

#### 2.2.1 5‐year RS definition

The 5‐year relative survival of colorectal cancer patients is defined as the observed survival of the patient group divided by the expected survival of the general population. The relative survival estimator uses the the Ederer II method according to according to Jiang's paper.^9^

#### 2.2.2 Period analysis calculated 5‐year RS from 2014 to 2018

The research patients included some patients who were diagnosed in 2014–2018 and those, and other patients who were diagnosed in 2009–2013 and survived in 2014–2018 were included from four cancer registries in Taizhou. The follow‐up period was 2014–2018. According to Brenner's study, the research cases were divided into patients newly diagnosed in the period of interest and patients diagnosed before the period of interest but still alive in the period of interest. Finally, the 5‐year relative survival was calculated.

#### 2.2.3 Model‐based period analysis predicted 5‐year RS from 2019 to 2023

This linear model, called model‐based period analysis was used to predict the 5‐year survival of colorectal cancer patients from 2019 to 2023 and was based on period analysis and made full use of the cancer registry data before December 31, 2018. First, the confirmed cases in 2004–2008, 2009–2013 and 2014–2018 were included according to the principles of the period analysis method. Then, calculated the number of exposures and deaths in each year, and calculated the conditional 1‐year survival in each year; Finally, the conditional 1‐year survival of each year was used as the dependent variable to fit the regression model (Poisson regression or binomial regression) with the follow‐up period and year as independent variables.

The conditional 1‐year survival *r* at the *j* period of follow‐up in *i* year after diagnosis is expressed as:

$$r_{\mathrm{ij}}=\exp\;\left(-\exp\left[\alpha_{i}+j\times \beta \right]\right).$$

where *j* represented the follow‐up period, *j* = 0 represents 2004–2008, *j* = 1 represents 2009–2013, and *j* = 2 represents 2014–2018. *I* represents the number of years of follow‐up in each period. For example, from 2004 to 2008, *i* = 12,004, *i* = 2, 2005, and so on.

The cumulative 5‐year relative survival of follow‐up in the *j* period is expressed as:

$$R_{j}=\Pi r_{\mathrm{ij}}=\text{Πexp}\;\left(-\exp\left(\alpha_{i}+j\times \beta \right)\right).$$

All statistical analyses were implemented with the “periodR” software package of R version 3.13 (R Foundation for Statistical Computing).

## 3 RESULTS

### 3.1 Basic characteristics of the CRC patients

The basic characteristics about CRC patients from 2004 to 2018 were shown in Table 1. Overall, 8486 cases were analyzed in the study, including 4987 male and 3499 female patients, respectively. The average diagnosed age was 64.6 years old. There were 548, 1125, 2178, 2351 and 2284 patients with diagnosed age at <45, 45–54, 55–64, 65–74 and >74 years old, respectively. Moreover, the number of the urban patients and rural patients was 1512 and 6974, respectively.

**TABLE 1:** Basic characteristics of colorectal cancer patients from Taizhou City, eastern China (2004–2018)

	Number of cases	Diagnosed interval		
		2004–2008	2009–2013	2014–2018
Gender				
Male	4987	423 (8.48%)	1689 (33.87%)	2875 (57.65%)
Female	3499	269 (7.69%)	1165 (33.30%)	2065 (59.02%)
Region				
Urban area	1512	45 (2.98%)	528 (34.92%)	939 (62.10%)
Rural area	6974	647 (9.28%)	2326 (33.35%)	4001 (57.37%)
Average age (years)	64.6	63.4	65.6	66.9
Age at diagnosis (years)				
<45	548	61 (11.13%)	213 (38.87%)	274 (50.00%)
45–54	1125	89 (7.91%)	389 (34.58%)	647 (57.51%)
55–64	2178	162 (7.44%)	718 (32.97%)	1298 (59.60%)
65–74	2351	215 (9.15%)	764 (32.50%)	1372 (58.36%)
>74	2284	165 (7.22%)	770 (33.71%)	1349 (59.06%)
Total	8486	692 (8.15%)	2854 (33.63%)	4940 (58.21%)

### 3.2 Five‐year relative survival estimated by period analysis for 2014–2018 period

As shown in Table 2, according to the data of four cancer registries in Taizhou City, the 5‐year RS of patients was 78.8% in the period of 2014–2018. According to gender, age of diagnosis and region, further stratified analysis was conducted. The 5‐year RS of male and female patients were 74.9% and 86.1%, respectively. The 5‐year RS of <45, 45–54, 55–64, 65–74 and >74 years old were 84.1%, 80.6%, 75.9%, 67.4% and 48.9%, respectively. Moreover, the 5‐year RS of urban and rural patients were 83.9% and 75.8%, respectively.

**TABLE 2:** The 5‐year RS of colorectal cancer patients during 2014–2018 and during 2019–2023

	2014–2018 5‐year RS (% ±SE)	2019–2023 5‐year RS (% ±SE)
Total	78.8 ± 0.7	85.9 ± 1.2
Gender		
Male	74.9 ± 0.4	79.1 ± 0.9
Female	86.1 ± 0.6	87.7 ± 1.3
Age at diagnosis (years)		
<45	84.1 ± 1.4	88.9 ± 1.6
45–54	80.6 ± 1.8	86.2 ± 0.9
55–64	75.9 ± 1.2	81.8 ± 2.0
65–74	67.4 ± 0.8	72.6 ± 1.1
>74	48.9 ± 0.9	53.4 ± 0.8
Region		
Urban area	83.9 ± 2.1	89.3 ± 2.3
Rural area	75.8 ± 0.8	80.2 ± 0.9

Abbreviations: RS, relative survival; SE, standard error.

### 3.3 Prediction of the 5‐year relative survival in 2019–2023

The 5‐year RS of CRC patients in 2019–2023 is estimated to be 85.9% (Table 2), 79.1% for men and 87.7% for women. The predicted 5‐year RS among patients aged <45, 45–54, 55–64, 65–74 and >74 years old were 88.9%, 86.2%, 81.8%, 72.6% and 53.4%, respectively. The 5‐year RS of urban and rural patients were 89.3% and 80.2%, respectively.

### 3.4 Trend of the 5‐year relative survival in four periods

**FIGURE 1:**
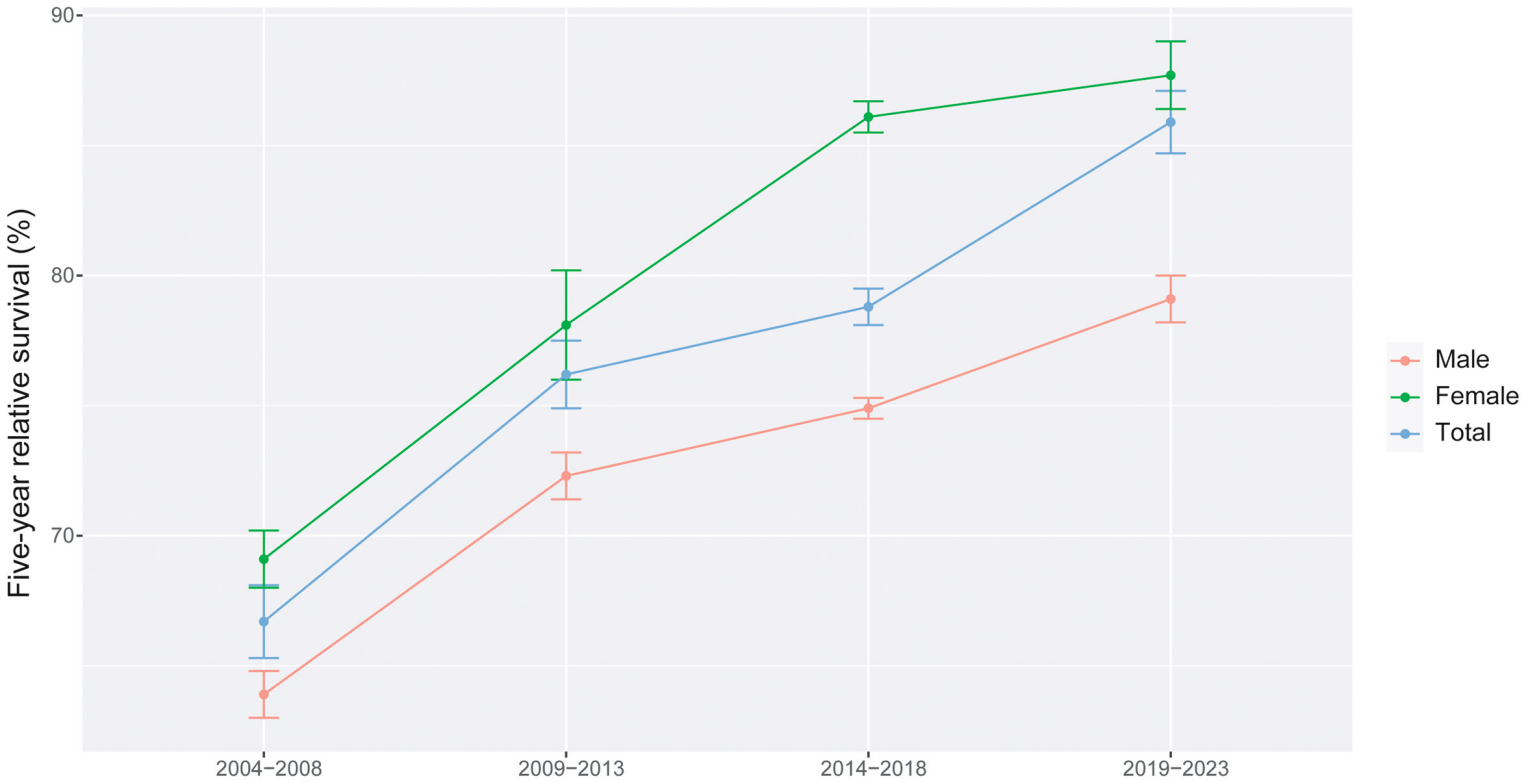
The 5‐year relative survival of colorectal cancer patients stratified by sex during 2004–2023.

**FIGURE 2:**
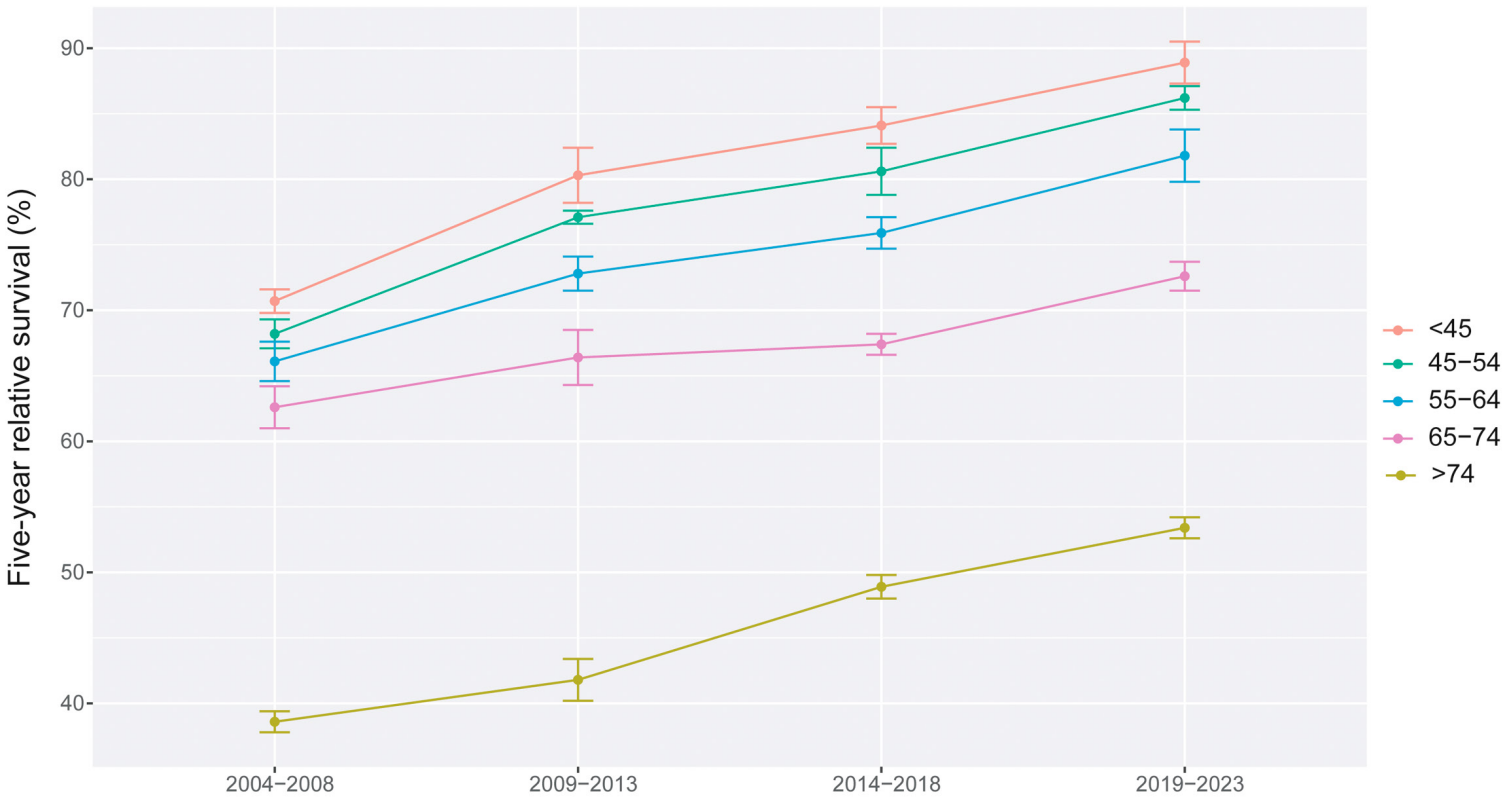
The 5‐year relative survival of colorectal cancer stratified by age at diagnosis during 2004–2023.

**FIGURE 3:**
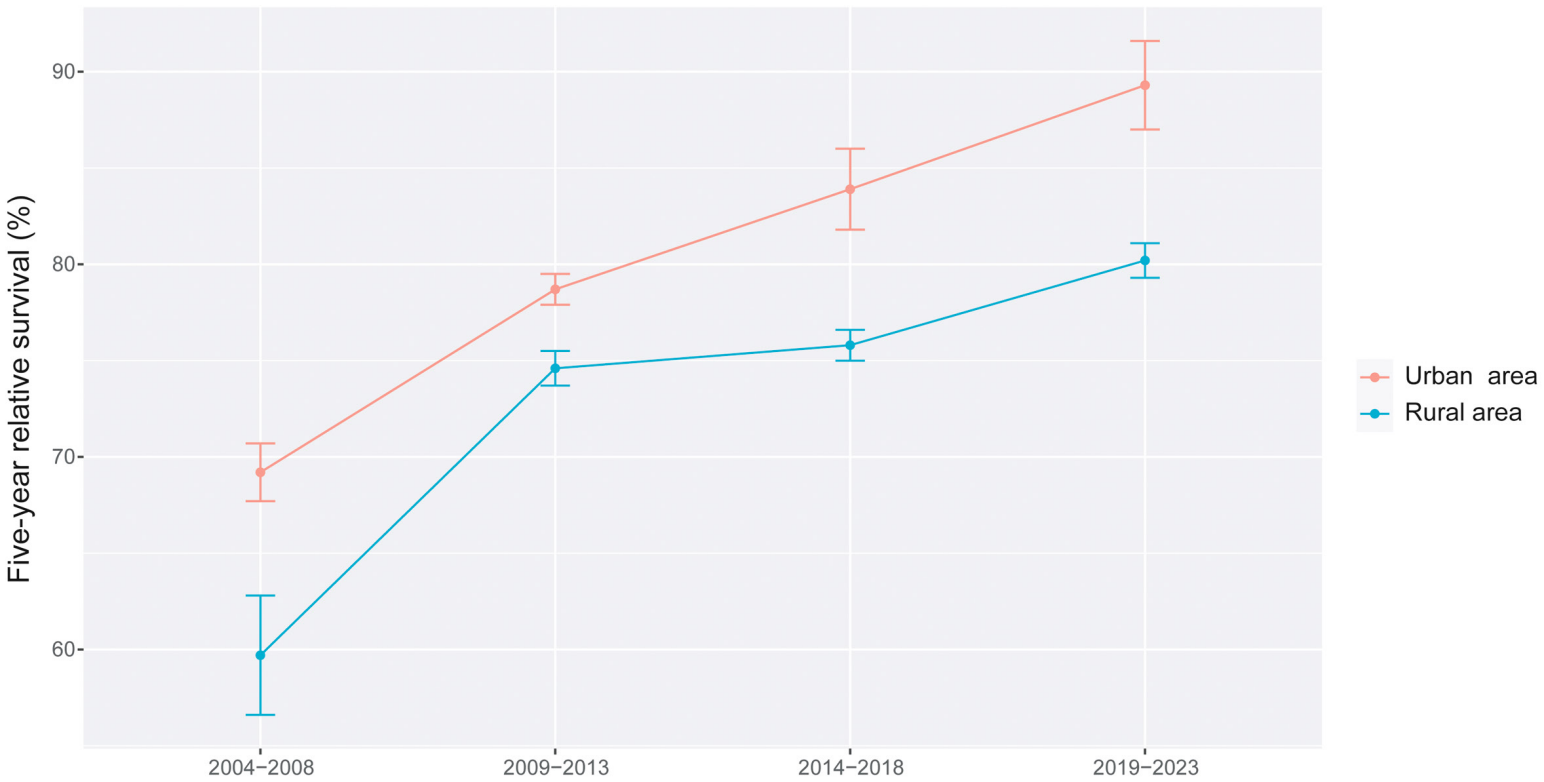
The 5‐year relative survival of colorectal cancer patients stratified by regions during 2004–2023.

In colorectal cancer patients, the overall 5‐year RS showed an increasing trend in 2004–2008, 2009–2013, 2014–2018 and 2019–2023. The survival of female patients was still higher than that of male patients. The 5‐year RS in each age‐stratification showed an upward trend in the four periods, and among patients under 45 years old was highest than other age groups. The survival gap between urban areas and rural areas was relatively stable, except for the period 2009–2013 (Figures 1, 2, 3).

## 4 DISCUSSION

The period analysis is a new method to evaluate long‐term survival timely and accurately. Our previous study verified that the period method was closer to the real survival than the traditional methods, with smaller deviation and standard error.^9^ In order to reduce the underestimation of survival due to the delay of the public cancer registry data, Brenner^10^ proposed a model‐based period method to avoid loss of precision and improve the estimates. In this study, we used the model‐based period method to evaluate 5‐year RS in Taizhou City.

The proportion of DCO below 13% indicates a good quality of cancer registry, which was recommended directly by Brenner when the corresponding author worked with him in Germany. Therefore, in this study the overall proportion of DCO cases less than 13% (from 9.8% to 12. 1%) was met for each recruited four cancer registries (Luqiao, Wenling, Xianju and Yuhuan). The proportion of DCO cases increased with aging, resulting in worse survival estimates. Therefore, high proportion of DCO cases had lower survival estimates, compared to low proportion of DCO cases.

In four periods, the 5‐year RS showed an upward trend, indicating a good prognosis trend for CRC patients in Taizhou City. We speculate that the early screening, improved treatment and expanded medical insurance for CRC may be the main reasons for the increasing survival in Taizhou City. First of all, the screening for CRC carried out in China since 2005, and the effect was obvious.^11^ Zhejiang Province has been carrying out cancer screening and early diagnosis and treatment in 1970s, which has got some achievements.^12^ According to the “Technical Plan for Early Diagnosis and Early Treatment of CRC” in China, a survey of risk assessment questionnaire and fecal occult blood test (FOBT) are combined to conduct preliminary CRC screening, and the high‐risk groups with positive results are further examined by colonoscopy. The target population of CRC screening in Zhejiang Province is local residents aged 40–74. Almost all eligible people were offered. FOBT is a simple screening method for CRC, which is suitable for rural areas, low‐income groups and people with poor compliance for colonoscopy. In China, the positive rate of FOBT among screened people was 14%, but in other Asian countries, the positive FOBT rate was 13.1%.^13^

Because of lack of specificity and low sensitivity in the diagnosis for CRC, FOBT can only be used for the initial screening and follow‐up population. Colonoscopy is the most intuitive and effective way for CRC screening in Chinese high‐risk areas, which is conducive to early detection of CRC and precancerous lesions. From a screening program for Zhejiang urban residents in 2013–2018, the colonoscopy compliance rate was 20%, and the detection rate for CRC was 0.3%.^14^ Screening can significantly improve early CRC detection and early surgical treatment, which prolongs the survival. There is a 2‐year overall survival rate of 95% in the CRC screening group and 80% in the non‐screening group after surgery.^15^

Serum tumor biomarkers are also important indicators for CRC screening. Carcinoembryonic antigen (CEA) is the commonly used biomarker. In order to improve the sensitivity and specificity of CRC screening, CEA is often combined with other biomarkers, such as neuron‐specific enolase (NSE), cancer antigen (CA) 199, CA125 and CA242.^16^ Recently, it has been reported that some biomarkers are closely related to the prognosis of CRC. High expressions of SAR1B (secretion associated Ras related GTPase 1B),^17^ HOXA11‐AS (homeobox A11 antisense RNA),^18^ or TRIM24 (tripartite motif containing 24)^19^ in CRC samples had the correlation with poor prognosis and shorter overall survival. Meanwhile, low expression of ENO2 and ENO3 were correlated with longer overall survival.^20^ According to reports, small molecule RNA (miRNA) is great promising biomarkers for CRC screening in China. The miRNA panel is superior to the FOBT and CEA measurement with higher sensitivity and specificity. The miRNAs detection, FOBT, CEA measurement can be used as a combined application for early CRC Screening.^21^ In addition, methylated DNA biomarkers are also potential biomarkers for early detection of CRC. Aberrant DNA methylation of SEPT9 and SDC2 demonstrated high sensitivities (89.1%) and specificity (90.8%) in stool specimens for early CRC detection.^22^ Because biomarkers are low‐cost, efficient and convenient, they are expected to become an ideal tool for early screening of CRC in China.

Second, the treatment for CRC is constantly updated. Surgical resection is the first choice, supplemented by radiotherapy, chemotherapy and traditional Chinese medicine. Early CRC can be treated by endoscopic treatment, which has achieved good results, especially for lesions that are less than 2 cm and clear deep margins.^23^ There was no significant difference in 5‐year tumor‐free survival rate between Chinese endoscopic group and surgery group for the treatment of early CRC.^24^ It has been reported that the 5‐year RS of patients with early CRC with onset of age < 50 years old can reach 96.7%.^25^ Even CRC patients over 80 years old have reported that the 5‐year overall survival rate after surgery can reach 36%.^26^ In recent years, colorectal surgery in China has developed rapidly due to the development of minimally invasive surgical technology, perioperative comprehensive treatment and clinical surgical technology research.^27^ Laparoscopic surgery is a common minimally invasive surgery with safer operation and fewer complications. Laparoscopic surgery and open surgery have no significant difference in recurrence and survival during follow‐up.^28^ Robotic surgery, another minimally invasive surgery, for treating CRC continues to increase. The robot surgery overcomes the limitations of laparoscopic surgery to precisely do pelvic floor surgery. Rectal cancer patients received robotic‐assisted transanal total mesorectal excision followed up for a median of 15 months, and there were no reports of recurrence, metastasis or death.^29^ Despite the high cost of robotic surgery, such surgery in China has developed rapidly.

Third, in China, since the reform of the medical system in 2009, effective strategies have been implemented to expand medical insurance. In recent years, affordable medical services have been available in both urban and rural areas,^11^, ^12^ and early treatment, early detection and early treatment of patients have improved the survival for CRC patients.

In this study, the survival of patients with CRC was further stratified by gender, age at diagnosis, and region. The 5‐year RS of male patients was lower than that of female patients. But the survival gap between male and female patients would shrink in predicted 2019–2023. Numerous studies have proved that both in China and abroad, the incidence and mortality rates of male CRC patients are higher than those of female patients, and the 5‐year survival of male CRC patients is slightly lower than female patients.^5^ In addition to greasy meals and obesity are closely related to the incidence of CRC, smoking proves to be one of risk factors of CRC, and the incidence of CRC increases linearly with smoking intensity and duration. In 2013, the male smoking rate in China was 47.2%, and far larger than female smokers (2.7%).^30^ Compared with smokers, never smokers and former smokers have a better prognosis for CRC with the higher overall survival.^31^ In addition, the drinking rate in Chinese adult men is also higher than Chinese adult women. The relationship between alcohol consumption and colorectal cancer risk has also been confirmed.^31^ These may be the reasons why the survival of male patients with CRC is lower than that of female patients. Therefore, quitting smoking and drinking as soon as possible may reduce the incidence and improve the survival for CRC patients, especially for male patients.

This study showed that the number of CRC patients living in rural areas was more than that in urban areas in Taizhou City. However, the 5‐year RS of urban areas was higher than rural areas. In rural areas, the lower medical care, low‐income and lower education level among patients compared with urban areas might be reasons for the low survival in the past few years.^32^ Because of late detection and late treatment, the survival in rural areas is lower than that in urban areas. Even in developed countries, the 5‐year survival of rural male patients with CRC is lower than that of urban male patients.^33^ But the prediction model showed that the rural survival dramatically improved in 2019–2023, indicating that the increased income, improvement of health awareness, and the improvement of medical and health construction have a positive impact on rural survival.

From the age at diagnosis stratification, the survival for each diagnosed age interval was increasing over time. The 5‐year survival of CRC patients under 45 years old had increased significantly. It has been reported that the cure rate of CRC patients younger than 50 years old is 65%, and the survival is significantly higher than that of patients over 50 years old.^34^ The survival of CRC patients any interval also greatly improved in predictive model, indicating that the current education intervention, screening and treatment strategies for CRC in Taizhou City will be effective for people of all age groups.

Our study has a number of strengths and limitations. Three strengths are listed as below. First, we provided, for first time in China using period analysis, most up‐to‐date (during 2014–2018) 5‐year RS for CRC patients from Taizhou, eastern China. Second, we assessed the survival trends and found 5‐year RS for CRC patients have improved greatly during 2004–2008, 2009–2013 and 2014–2018. Third, we projected the upcoming 5‐year RS during 2019–2023. We also have limitations. First, we could not provide stratified survival data on stage, histology and treatment for CRC patients. Nevertheless, population‐based cancer registries commonly do not include clinical information on stage (such as TNM), histology and treatment of cancer patients. Hopefully hospital‐based cancer registries including detailed information on cancer patients will be available soon in the near future. Second, we only provided most up‐to‐date survival data for CRC patients from Taizhou, eastern China. In addition, the generalized linear model has some limitations. When the trend of survival does not change evenly over time, tends to decrease, survival may be overestimated. Conversely, when survival rates increase significantly over time, survival may be underestimated. Therefore, further investigations using provincial or national data are also highly warranted.

In conclusion, we provided, for first time in China using period analysis, most up‐to‐date 5‐year RS for patients with CRC from Taizhou, eastern China and also found 5‐year RS for CRC patients have improved greatly during 2004–2018.

## AUTHOR CONTRIBUTIONS

**Min Zhang:** Writing – original draft (lead). **Yongran Cheng:** Formal analysis (lead); software (equal). **Bicheng Chen:** Conceptualization (equal); writing – original draft (equal); writing – review and editing (equal). **Runhua Li:** Investigation (equal). **Xiyi Jiang:** Data curation (equal); software (equal). **Liangyou Wang:** Data curation (lead); validation (equal). **Tianhui Chen:** Conceptualization (lead); funding acquisition (equal); writing – review and editing (lead). **Qi Liao:** Funding acquisition (lead); writing – review and editing (equal). **Jinfei Chen:** Conceptualization (equal); funding acquisition (lead); writing – review and editing (equal).

## CONFLICT OF INTEREST

None.

## ETHICAL STATEMENT

Although the data from nine cancer registries from Taizhou City, eastern China were completely anonymous and their use did not entail ethical problems, ethical approval by the Institutional Review Board of Zhejiang Cancer Hospital, China was obtained.

## Data Availability

The data supporting our results can be obtained at the reasonable request from the corresponding authors. The data are not publicly available due to privacy or ethical restrictions.
